# Prevalence of Diabetes Mellitus among Roma Populations—A Systematic Review

**DOI:** 10.3390/ijerph15112607

**Published:** 2018-11-21

**Authors:** Marisa A. Nunes, Kristýna Kučerová, Ondřej Lukáč, Milan Kvapil, Jan Brož

**Affiliations:** 1 Department of Internal Medicine, Second Faculty of Medicine, Charles University, 150 00 Prague 5, Czech Republic; marisa_nunes.9@hotmail.com (M.A.N.); lukaco@seznam.cz (O.L.); milan.kvapil@fnmotol.cz (M.K.); 2 Arbeitsgemeinschaft der Belegärzte am Alice-Hospital, 64287 Darmstadt, Germany; kristyn.kucerova@gmail.com

**Keywords:** diabetes mellitus, prevalence, Romani, Roma ethnic, gypsy

## Abstract

Background: The aim of this study was to estimate the prevalence of diabetes mellitus in the Roma population and compare it to the prevalence in the Caucasian population. Methods: Using the words “Roma”, “Gypsies”, “Romani”, and ”traveler” in combination with “diabetes, “metabolic syndrome”, “cardiovascular disease” and “health status” we searched the MEDLINE, Pubmed and Scopus databases for articles in English that focused on the prevalence of diabetes mellitus among Roma populations published until December 2017. Results: Five studies met the inclusion criteria. The results of four of them suggested a higher prevalence of diabetes among Romani compared to Caucasians but none of them reached the standards regarding representative samples and number of cases for a conclusive result. Conclusion: Although some of the existing studies suggest a substantial prevalence of diabetes among Roma populations and even a higher risk of developing diabetes for Roma persons compared to Caucasians, the number of published literature on this topic remains very low and insufficient in design and number of participants to draw any conclusions.

## 1. Introduction

The number of people with diabetes mellitus is increasing worldwide [1]. Diabetes prevalence differs among countries, but also among different ethnic groups [2,3].

The Roma constitute the largest minority group in Europe [4] and have an average estimated population of just over 11 million, most living in Central and Eastern Europe [5]. The exact population size remains largely unknown due to lack of official documentation and a fear of stigmatization, resulting in their reluctance to identify themselves [6].

Many studies report health inequality, poor living conditions and other social determinants of health such as low level of education, social exclusion, a high rate of unemployment, poor nutrition, and low socio-economic status in Roma populations [7,8,9,10,11,12]. Studies also report a shorter life expectancy among Roma than among the majority population, with a mainly progressive population pyramid characterized by a large percentage of younger people and a low density of elderly people [13], and a higher prevalence of communicable and non-communicable diseases [4,14]. Several studies have suggested a significantly higher prevalence of metabolic syndrome among Roma populations [8,15,16].

With the aforementioned status of public health and especially regarding the suspected high prevalence of metabolic syndrome, it is appropriate to consider their impact also on the prevalence of diabetes in the Roma population.

This study aims to give a comprehensive review of the existing literature on diabetes prevalence in the Roma population. Additionally, information on factors associated with diabetes assessed in the studies is mentioned.

## 2. Material and Methods

### 2.1. Data Search

A literature search conducted in December 2017 using Scopus, PubMed and Medline on articles published until December 2017 utilized the following keywords: “Roma”, “Gypsies”, “Romani”, and ”traveler”, in combination with “diabetes, “metabolic syndrome”, “cardiovascular disease” and “health status”. This search revealed a variety of publications published since 1970, including 256 articles, reviews, letters and conference papers. An additional four publications were identified through “suggested reading”, which were then manually assessed for relevance to diabetes. Duplicate data were excluded, as well as any publication not written in English.

This review is reported in accordance with the PRISMA statement [17] for the reporting of systematic reviews and meta-analyses. No previous review of literature on diabetes prevalence among the Romani population was found. The results are listed in reverse chronological order according to the year of publication. The basic characteristics of the studies are shown in Table 1.

### 2.2. Inclusion Criteria

Only studies directly investigating the prevalence of diabetes were included in this analysis. Studies conducted since 1999 had to follow the definition of diabetes mellitus according to the 1999 WHO diagnostic criteria (fasting plasma glucose (FPG) ≥7.0 mmoL/L, random plasma glucose ≥11.1 mmoL/L or plasma glucose 2-h post-glucose load (OGTT) ≥11.1 mmoL/L) [25], and those published prior to 1999 according to the 1985 WHO definition (fasting plasma glucose ≥7.8 mmoL/L or or plasma glucose 2-h post-glucose load ≥11.1 mmoL/L) [26]. Only articles written in English were considered.

### 2.3. Compliance with Ethics Guidelines

This review is based on previously conducted studies and does not contain any studies with human participants or animals performed by any of the authors.

## 3. Results

**Figure 1:**
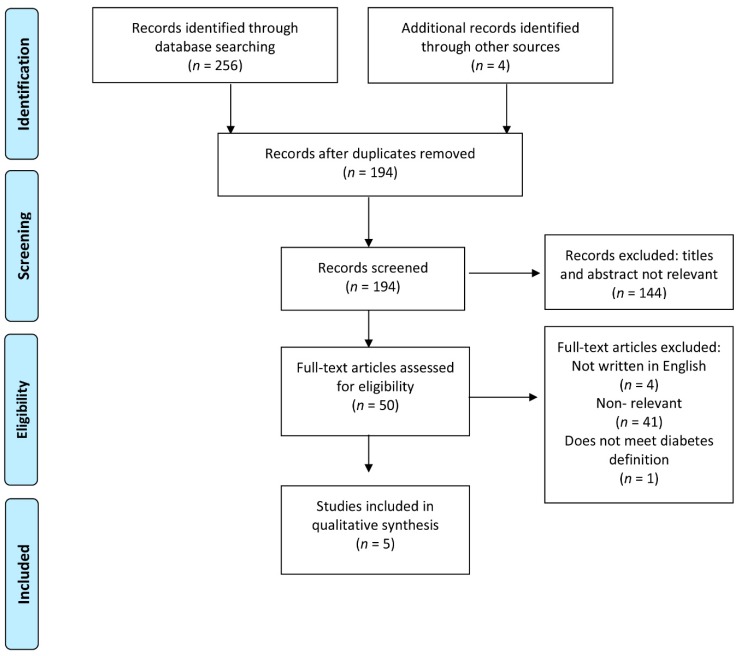
Flow diagram.

Only four articles focused primarily on diabetes prevalence, and one article was identified that focused primarily on metabolic syndrome prevalence and also on the prevalence of diabetes in the Roma population (Figure 1).

Enache et al. analyzed the records of 180 Roma (61 males) and 164 non-Roma (56 males) with the aim of assessing the prevalence of newly diagnosed diabetes in the Roma population of one county in Romania. The type of diabetes was not specified. The results showed the presence of diabetes in 11.7% of Roma people and in 14.6% of Romanian Caucasians, but the difference was not statistically significant. Additionally, the study focused on the evaluation of obesity prevalence. A high prevalence of obesity in Roma people but also in the non-Roma population (31.7% vs. 32.3%) and overweight subjects (26.7% vs. 37.2%) was observed; however the statistical significance was not calculated. It was found that 72.8% (*n* = 131) of Roma persons and 80.5% (*n* = 132) of Romanian Caucasians presented central obesity based on waist circumference measurement. Hypertension was significantly more prevalent in Romanian Caucasians (64% (*n* = 105) vs. 48.9% (*n* = 88)). Data related to physical activity, food consumption and smoking were obtained using the Finnish Diabetes Risk Score questionnaire [27]. Based on these results, the risk factors for obesity in the Roma population were: lower socio-educational level, smoking, and physical inactivity (active less than 30 min/day). The prevalence of cigarette smoking was statistically significantly higher in the Roma population than in the majority population (45.6% vs. 23.1%). The study’s limitations are the low number of participants and the non-inclusion of subjects with previously diagnosed type 1 (*n* = 2) and type 2 diabetes (*n* = 58) in the study group, without clearly indicating the percentage of Roma vs. non-Roma present [18].

Živković et al. assessed members of the Gypsy community from 8 rural and 11 urban settlements in Serbia for the prevalence of diabetes. The type of diabetes was not specified. The authors, from a planned sample size of 2000 patients, evaluated the complete health reports of 1465 participants (953 female and 512 male) in the following age groups: 18–35 years old (28.9%), 35–54 years old (38.2%), 54–64 years old (27.4%), and older than 65 years (5.6%). Of the participants with previously diagnosed diabetes (5.9%), all but two had type 2 diabetes mellitus. The study also revealed 5.2% of participants had newly diagnosed cases of type 2 diabetes with the average age of the newly afflicted participants being from 50.4 ± 17.2 years old. In 72% of the studied Roma population no diabetes was identified, but the results of 17.3% of subjects were ambiguous. In the case of a positive family history, the risk of developing diabetes was 3.48 times higher than in participants with a negative history (95% confidence interval (CI), *p* < 0.01), and the evaluated risk of developing diabetes in participants with abdominal obesity was twice as high (95% CI, *p* < 0.01). Diabetes was 3.65 times more present in urban populations as compared to those living in rural settlements. The study’s limitation is its non-representative population sample and the absence of the majority population group [19].

Vozárová de Courten et al. conducted a study in a small village in Slovakia with 1800 inhabitants (40% Romani). In general, participating Roma subjects were younger (47 vs. 52 years, respectively), heavier (87 vs. 78 kg), and had a higher BMI (32 vs. 28 kg/m^2^) and waist-hip ratio 0.91 vs. 0.87) when compared with non-Roma participants (all *p* < 0.0001). Romani subjects also reported higher smoking rates (42% vs. 21%, respectively, *p* < 0.001), less physical activity (3% vs. 14%, *p* < 0.001) and lower levels of education (24% and 58%, *p* < 0.0001 for high school or university education) than non-Romani subjects. Thirty-eight percent of the Roma population was unemployed and 30% had a monthly income less than 2000 Slovak crowns (equivalent of US$40) compared with 5% and 4% of non-Romani subjects (both *p* < 0.001), respectively. Romani subjects showed the age- and sex-standardized three times higher and a statistically significant prevalence of Type 2 diabetes accompanied by more than twice the prevalence of obesity as well as higher prevalence rates of hypercholesterolemia, hypertriglyceridemia, hyperinsulinemia, insulin resistance, elevated microalbuminuria, metabolic syndrome and cardiovascular disease. There were, however, similar rates of hypertension. Additionally, a higher insulin resistance and obesity prevalence was found in Roma people without diabetes mellitus compared to Caucasians. The study’s limitation is the low response rate of 53% for non-Roma (230 male/271 female) and 28% for Romani persons (70 male/86 female) [21].

Thomas et al. published a study conducted in Boston with a sample size of 58 Roma persons of ages between 16 and 72 years and found the presence of diabetes in 46% of individuals, hypertension in 73%, hypertriglyceridemia in 80%, hypercholesterolemia in 67%, occlusive vascular disease in 39%, and chronic renal insufficiency in 20%. The type of diabetes was not specified. Furthermore, negative health habits were observed, where 86% of the individuals smoked cigarettes and 84% were obese (defined as more than 20% over the ideal weight). Of all analyzed studies, this was the only article which used the older criteria for diabetes diagnosis (fasting plasma glucose ≥7.8 mmoL/L). Besides the non-representative population sample, another limitation is that most of the subjects were members of the same family-39 subjects were descended from the same ancestor, and 15 were connected to the family by marriage and therefore were “distant cousins” [22].

Hidvegi et al. conducted a study in the city of Győr and surrounding area, screening 77 Gypsy individuals (35 men, 42 women; age: 46.9 ± 10.6 years) and revealed 14 cases of newly diagnosed type 2 diabetes mellitus (18.2%) with a higher frequency than that of the representative population in Hungary (7.47%). An additional 14 cases of pre-diabetes (18.2%) were encountered. The study’s limitation is the low number of participants and the absence of the majority population group [23].

## 4. Discussion

Out of all the studies we found, only two looked at the prevalence of type 2 diabetes mellitus (T2DM) separately, with the remaining three articles not specifying any particular type of diabetes. With this in mind, it is possible that the prevalence of diabetes presented in the results consists not only of type 2 diabetes mellitus but of other specific types, such as those based on diseases of the exocrine pancreas, liver or even Maturity Onset Diabetes of the Young [28,29,30]. However, as T2DM accounts for more than 90 percent of all diabetes patients [31], it is still plausible to presume that the highest portion corresponds to T2DM.

Upon review of all the individual studies, and bearing in mind the various difficulties presented by such studies, the inability to reach a population representative sample coupled with the limitation in the number or rate of participants’ responses are evident and severely weaken the results.

The only study with a larger number of participants is the one from Serbia, but it did not reach the planned subject number, and was conducted only in selected regions of the country without a comparative or control group. The rest of the studies only dealt with a low number of subjects not exceeding 200 participants. Furthermore, control groups were only included in some studies [23,25] with the rest relying on previously acquired population data [18].

In spite of this, four out of five studies suggested a higher diabetes prevalence among Roma populations. In the study which showed no increased prevalence [23], it should be noted that individuals who had been previously diagnosed with diabetes were not included.

All these factors prevent us from reaching any conclusive findings about the risk of developing diabetes among Roma, and making a comparison with the risk in the Caucasian population. Based on the above mentioned suggestions, we may, however, attempt to speculate on possible explanations for the findings of a higher rate of type 2 diabetes in Roma populations.

In addition, some supporting evidence for these findings is provided by several studies focusing on metabolic syndrome [15,16,27], which, although also limited by the low numbers of participants, suggest its higher prevalence among Roma populations. As diabetes is a part of the metabolic syndrome, we may therefore infer that the prevalence of diabetes could also be higher.

There are several known main risk factors of type 2 diabetes: sedentary life style, unhealthy dietary habits, low socio-economic status, smoking, and alcohol consumption [32]. These factors contribute to a higher degree of obesity with higher insulin resistance (usually in the form of metabolic syndrome), which together with an inadequate beta cell response (due to β-cell dysfunction), results in loss of glycemic control and an increased risk of diabetes [33]. All risk factors were reported as present more regularly in the Romani population compared with the majority populations in some of the studies: smoking [34,35,36,37,38,39,40], sedentary life style [14,25,40,41], unhealthy dietary habits [39,40,42], low socio-economic status [6,25] and alcohol consumption [40,37], associated also with a higher incidence of obesity in many of them. However, all of these studies were again conducted on a rather small number of subjects.

It should also be stated that one of the studies [25] indicated the early manifestation of type 2 diabetes, suggesting that insulin resistance begins earlier in Roma populations. This conclusion is also supported by a small study showing significantly higher insulin resistance in young Roma compared with Slovaks of the same age [34] and in another study of the same authors showing higher insulin levels in Roma participants in BMI ≥25 [43]. These studies at least showed that any future screening for diabetes among Roma populations must be conducted among a younger age group than is usual among Caucasians, being sure to include those with a lower BMI.

Other than the previously mentioned “metabolic” risk factors, cultural and psychosocial factors may also play a role. Data from several countries in the European Union suggest that discrimination or fear of discrimination (self-assessed) or cultural barriers are preventing Roma people from accessing health care, with consequences such as poor access to preventive medicine. This may be another possible factor for the potentially higher prevalence of diabetes among Roma populations [44]. Also, a higher rate of unemployment among Roma people [44] compared to the general population can, as an indirect risk factor for developing diabetes, contribute to the higher diabetes prevalence [45,46].

Another important aspect generally seen in these communities which could contribute to these results is the lack of perception of health as an important cultural value [7]. Říčan stated that health occupies the 10th position in the Roma value chart, whereas in the majority population it occupies the first place [7,46,47]. This view on health can then have negative results on overall health status and could also, together with language and literacy barriers and lack of knowledge about their entitlements concerning welfare and available services, partly explain the unsatisfactory participation of Roma communities in preventive health programs [44].

We must also mention the results of the meta-analysis focused on type 2 diabetes prevalence among ethnic minority groups resident in Europe [48]. Twelve of the included articles presented data on South Asian populations, which could be considered as having roots in a similar part of the world as the Roma [49]. All found a higher type 2 diabetes prevalence among these populations when compared to their host European ones. When the data were pooled, overall, South Asians had 3.7 (95% CI 2.7–5.1) higher odds ratio for T2DM compared to their European host populations. Among them Bangladeshis had the highest odds ratio for T2DM, (6.2, 95% CI 3.9–9.8), followed by Pakistanis (5.4, 95% CI 3.2–9.3), and Indians (4.1, 95% CI 3.0–5.7) compared with Europeans [48]. A study conducted in Australia even showed a prevalence of type 2 diabetes several times higher among migrants from South and Central Asia than among the Australian-born population across all socio-economic strata, suggesting that socio-economic status may not be a key point for this risk increase [50]. It should also be mentioned that among lean, healthy individuals matched for age, BMI, waist circumference, birth weight, and current diet, Asians (especially those of Southeast Asian descent) had higher levels of postprandial glycemia and lower insulin sensitivity than Caucasians in response to a 75-g carbohydrate load [51]. These findings raise the possibility that Asians are more genetically susceptible to insulin resistance and diabetes than Caucasians. This possible genetic risk is supported by several studies [52,53,54,55,56,57,58] suggesting that beta cell dysfunction plays a critical and more important role in the development of diabetes in Asians [59].

Insofar as we may consider the Roma people to be members of a wider Asian ethnicity, we may cite the above-mentioned findings in support of the hypothesis that they are more at risk of developing diabetes. They left their home country centuries ago, but by keeping themselves rather separate from their host populations, they could have maintained their “diabetes risk” until today.

Irrespective of the comparison between Roma and non-Roma populations, the prevalence of diabetes among this minority group seems to be substantial. More attention should therefore be given not only to diabetes screening but also to educational and health promotion programs tailored to this ethnicity, which should be developed with the aim of reducing the heightened risk for diabetes.

## 5. Conclusions

Although some of the existing studies suggest a substantial prevalence of diabetes among Roma populations and even a higher risk of developing diabetes for Roma persons compared to non-Roma persons, the amount of published literature on this topic remains very low and insufficient in design and number of participants to draw any conclusions. Therefore, further investigation in this area is needed. Some findings testify that any future screening for diabetes among Roma populations should be conducted among a younger age group than is usual among Caucasians, and probably also with those with a lower BMI.

## Figures and Tables

**Table 1:** Basic characteristics of the analyzed studies.

Authors	Year of Publication of Study	Country	Subjects’ Age Range (Years)	Percentage of Females in Roma Study Population	Method of Diabetes Diagnosis	Prevalence of Diabetes in Roma Study Population, Number of Subjects	Prevalence of Diabetes in Non-Roma Study Population, Number of Subjects	Statistical Significance
Enache et al. [18]	2016	Romania, (Călăraşi county)	18–85	65.9	FPG or HbA1c *	11.7%, *n* = 180	14.6%, *n* = 164	Not significant
Živković et al. [19]	2010	Serbia, 11 urban and 8 rural settlements	≥18	65.1	FPG or random	11.1%, *n* = 1465	6.7% ** [20]	Not calculated
Vozárová de Courten et al. [21]	2003	Slovakia (Zlaté Klasy)	≥30	55.1	FPG	30.0%, *n* = 156	10.0%, *n* = 501	*p* < 0.0001
Thomas et al. [22]	1987	United States, (Boston). Mostly members of one family.	16–72	Not stated	FPG or HbA1c	46%, *n* = 58	11.4% * [1]	Not calculated
Hidvegi et al. [23]	2012	Hungary (Győr and surroundings)	20–70	54.5	FPG or OGTT	18.2%, *n* = 14	7.47% * [24]	Not calculated

* HbA1c = glycated hemoglobin; ** Study did not include subjects from the non-Roma population. The source of the population’s diabetes prevalence is given in the reference. FPG = fasting plasma glucose, OGTT = oral glucose tolerance test.
